# Zebrafish as an Animal Model for Drug Discovery in Parkinson’s Disease and Other Movement Disorders: A Systematic Review

**DOI:** 10.3389/fneur.2018.00347

**Published:** 2018-06-01

**Authors:** Rita L. Vaz, Tiago F. Outeiro, Joaquim J. Ferreira

**Affiliations:** ^1^TechnoPhage, SA, Lisboa, Portugal; ^2^Faculdade de Medicina, Universidade de Lisboa, Lisboa, Portugal; ^3^Department of Experimental Neurodegeneration, Center for Nanoscale Microscopy and Molecular Physiology of the Brain, University Medical Center Göttingen, Göttingen, Germany; ^4^Department of Experimental Neurodegeneration, Center for Biostructural Imaging of Neurodegeneration, University Medical Center Göttingen, Göttingen, Germany; ^5^CEDOC, Chronic Diseases Research Centre, Faculdade de Ciências Médicas, NOVA Medical School, Universidade NOVA de Lisboa, Lisboa, Portugal; ^6^The Medical School, Institute of Neuroscience, Newcastle University, Newcastle upon Tyne, United Kingdom; ^7^Faculdade de Medicina, Instituto de Medicina Molecular, Universidade de Lisboa, Lisboa, Portugal; ^8^Laboratory of Clinical Pharmacology and Therapeutics, Faculdade de Medicina, Universidade de Lisboa, Lisboa, Portugal; ^9^CNS-Campus Neurológico Sénior, Torres Vedras, Portugal

**Keywords:** drug discovery, hyperkinesia, hypokinesia, movement disorders, zebrafish models

## Abstract

Movement disorders can be primarily divided into hypokinetic and hyperkinetic. Most of the hypokinetic syndromes are associated with the neurodegenerative disorder Parkinson’s disease (PD). By contrast, hyperkinetic syndromes encompass a broader array of diseases, including dystonia, essential tremor, or Huntington’s disease. The discovery of effective therapies for these disorders has been challenging and has also involved the development and characterization of accurate animal models for the screening of new drugs. Zebrafish constitutes an alternative vertebrate model for the study of movement disorders. The neuronal circuitries involved in movement in zebrafish are well characterized, and most of the associated molecular mechanisms are highly conserved. Particularly, zebrafish models of PD have contributed to a better understanding of the role of several genes implicated in the disease. Furthermore, zebrafish is a vertebrate model particularly suited for large-scale drug screenings. The relatively small size of zebrafish, optical transparency, and lifecycle, are key characteristics that facilitate the study of multiple compounds at the same time. Several transgenic, knockdown, and mutant zebrafish lines have been generated and characterized. Therefore, it is central to critically analyze these zebrafish lines and understand their suitability as models of movement disorders. Here, we revise the pathogenic mechanisms, phenotypes, and responsiveness to pharmacotherapies of zebrafish lines of the most common movement disorders. A systematic review of the literature was conducted by including all studies reporting the characterization of zebrafish models of the movement disorders selected from five bibliographic databases. A total of 63 studies were analyzed, and the most relevant data within the scope of this review were gathered. The majority (62%) of the studies were focused in the characterization of zebrafish models of PD. Overall, the zebrafish models included display conserved biochemical and neurobehavioral features of the phenomenology in humans. Nevertheless, in light of what is known for all animal models available, the use of zebrafish as a model for drug discovery requires further optimization. Future technological developments alongside with a deeper understanding of the molecular bases of these disorders should enable the development of novel zebrafish lines that can prove useful for drug discovery for movement disorders.

## Introduction

**Figure 1:**
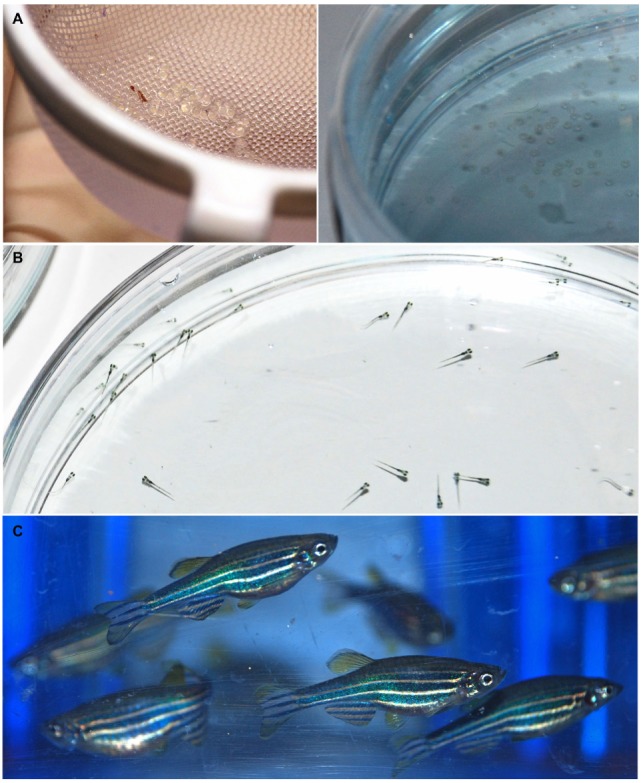
Representative images of zebrafish at embryonic **(A)**, larval **(B)**, and adult **(C)** stage.

Movement disorders are a heterogeneous group of neurological conditions characterized by the inability to produce or control movement. The typical clinical features include either paucity of voluntary movements, referred to as hypokinesia, bradykinesia and akinesia, or excess of movement, commonly denoted as hyperkinesia, dyskinesia, and abnormal involuntary movements (1). These two major groups have been dynamic, including different categories over time. Most movement disorders lack effective pharmacological therapies, because their complex etiology and pathological mechanisms remain largely unknown. This complicates the development of adequate animal models and, ultimately, of therapeutic compounds. In this context, zebrafish (*Danio rerio*) (Figure 1) has become an attractive tool for drug discovery. Zebrafish presents a compromise between the scalability of invertebrate models and overall homology to vertebrates. In the last 20 years, several zebrafish models of brain disorders have been generated (2). Many discoveries were reported, but an overall analysis of zebrafish as an alternative model of movement disorders is lacking. Therefore, the scope of this review was to systematically analyze the latest developments in the generation and characterization of zebrafish models of the most common movement disorders. This highlights the translational value of zebrafish to model these diseases and, ultimately, for drug discovery. The pathogenic mechanisms, disease hallmarks, phenotypic effects of mutations or neurotoxins, and responsiveness to pharmacological interventions are covered for zebrafish models of two hypokinetic and five hyperkinetic disorders (Table 1).

**Table 1:** Etiology, phenomenology, and pharmacotherapies of the movement disorders covered in this review.

	Disease	Etiology	Pathological hallmarks	Motor symptoms	Pharmacotherapy	Disease-modifying therapy	Reference
Hypokinesias	Parkinson’s disease	Environmental factors Genes: SNCA, leucine-rich repeat kinase 2 (LRRK2), DJ-1, VPS35, P1NK1, Parkin Genetic polymorphisms	Dopaminergic cell loss Lewy bodies	Bradykinesia Resting tremor Postural instability Muscular rigidity	**Levodopa** Dopamine agonists Monoamine oxidase inhibitors Catechol-*o*-methyl transferases inhibitors	**None** Isradipine^a^ Caffeine^a^ Nicotine^a^ Inosine^a^ Ab and vaccines^a^ Nilotinib^a^	(27)

	Progressive supranuclear palsy	MAPT polymorphisms (tau protein) Gene: MAPT Genetic risk factors: STX6, EIF2AK3, and MOBP	Diffuse neuronal loss (cortex, globus, pallidus, subthalamic nucleus, and substantia nigra) Neurofibrillary tangles or neuropil threads (with tau protein) in basal ganglia and brainstem	Unexplained falls Unsteady gait Bradykinesia Ocular motor deficits	**None** Levodopa (few) Taxane (microtubule-stabilizing drug) Tau Ab and vaccines Salsalate (tau acetylation inhibitor)	None	(114)

Hyperkinesias	Dystonia	Environmental factors Genes: TOR1A, THAP1, DYT13, DYT21, (GCH1), (SGCE), (ATP1A3, PRKRA, SLC6A3) Polymorphisms DYT7	Malfunction of basal ganglia Over-excitability of sensorimotor cortex	Excessive, uncontrolled muscle contractions Muscle spasms Abnormal posture	Levodopa (dopa-responsive dystonia) Dopamine agonists (dopa-responsive dystonia) **Anticholinergics** **Antidopaminergics** Clonazepam Acetazolamide Morphine sulfate Sodium oxybate Levetiracetam Zonisamide	None	(118–120)
	
	Chorea in Huntington’s disease	Gene: huntingtin (HTT)	GABAergic MSN loss Intranuclear inclusions of HTT	Chorea Dystonia Bradykinesia Incoordination	**Tetrabenazine** (VMTA2 inhibitor) Haloperidol Fluphenazine Olanzapine	**None** Coenzyme Q10^a^ Creatine^a^ Dimebon^a^ Ethyl eicosapentaenoate (Miraxion)^a^ Minocycline^a^ Ab and vaccines^a^	(120, 134)
	
	Stereotypies in Rett syndrome	Gene: MECP2	Reduction of brain volume Abnormally small, densely packed neurons with reduced dendritic complexity and synapse density	Loss of hand skills Gait abnormality Postural instability Hand stereotypies	**None** Glutamate modulators (dextromethorphan and ketamine)^a^ Monoamine modulators (desipramine and sarizotan)^a^ Neurotrophic factors (fingolimod, mecasermin, and trofinetide)^a^ Metabolic factors (lovastatin)^a^ Modulators of mitochondrial function (EPI-743 and triheptanoin)^a^	None	(155)
	
	Essential tremor	Unknown genetic and environmental factors	Potentially abnormal cerebellar-thalamic outflow pathways	Progressive active tremor	**Propranolol** (β-adrenergic blocker) **Primidone** (anticonvulsant)	None	(120, 169, 170)
	
	Tics in Tourette’s syndrome	Environmental factors Risk genetic polymorphisms	Potentially impaired cortico–striato–thalamocortical circuits	Chronic motortics	**Dopamine receptor blocking drugs** (fluphenazine, haloperidol, and risperidone) **Monoamine-depleting drugs** (tetrabenazine)	None	(172)

*^a^Clinical trails*.

*Bold: gold standard pharmacotherapy*.

### Zebrafish as a Model for Translational Research

**Figure 2:**
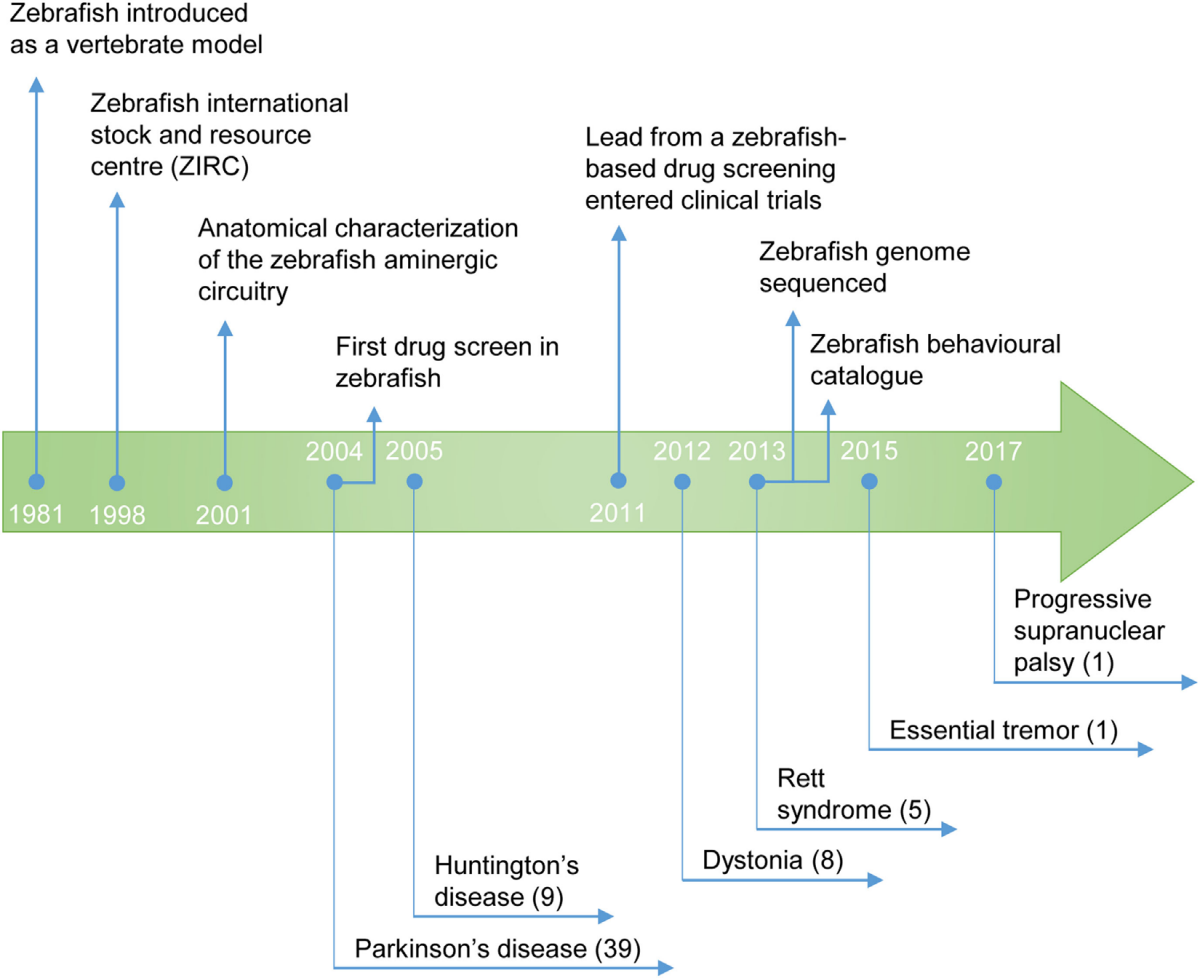
Timeline of the use of zebrafish as a model for the study of movement disorders and drug discovery. The publication year of the first study describing a zebrafish model of the movement disorder is highlighted. (#) Number of studies published to date.

**Figure 3:**
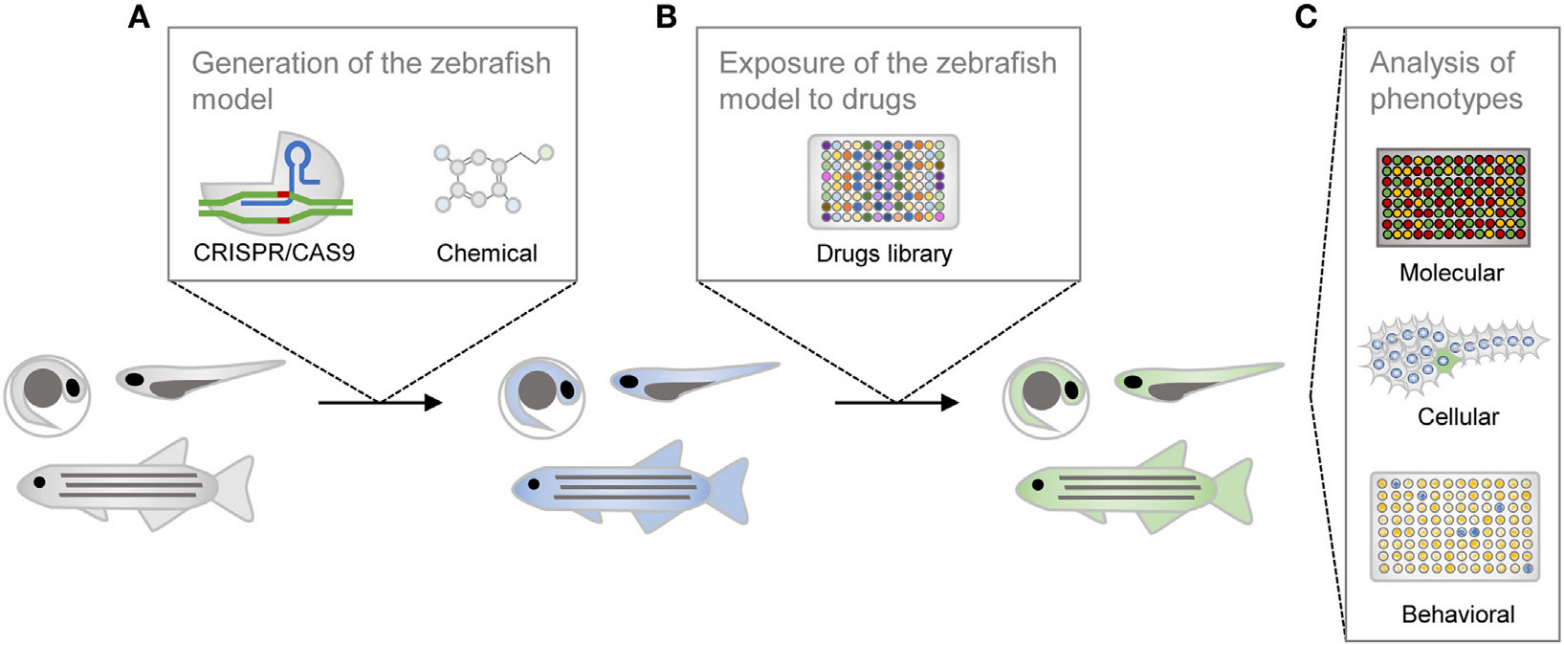
Schematic representation of the drug screening process in zebrafish. The zebrafish line is generated with genetic or chemical tools **(A)**, is incubated with compounds from the library of small molecules **(B)**, and is then phenotypically characterized **(C)**.

The utilization of zebrafish for drug discovery increased in the beginning of the twenty-first century (Figure 2) (3, 4). Due to its small size and fast reproduction, zebrafish is suitable for large-scale *in vivo* assays. Drug administration is facilitated through the aqueous environment, and the efficacy, bioavailability and toxicity can be readily determined. Importantly, zebrafish is a vertebrate, in contrast to other commonly used organisms, such as *Drosophila melanogaster* or *Caenorhabditis elegans*, in which the anatomical similarity with humans is much lower (5). The optical transparency is another advantage of this teleost, as it enables the direct observation of cellular and physiological processes *in vivo* and in real time. These and other practical features rendered zebrafish the mainstream model for investigation in developmental biology. In addition, it is now also widely used as a disease model and, more recently, it became an important tool for the screening of drugs (Figure 3) (6).

Despite the evident differences between fish and mammals, zebrafish hold genomic and physiological homology to humans (7). Moreover, the genome of zebrafish is sequenced and available for annotation in databases. The genome of zebrafish includes orthologs of 71% of human genes, and a high degree of conservation in the functional properties of many of the encoded proteins (8). Physiological and anatomical homology is also evidenced in most of the organs, including the nervous system (7). The basic anatomical structure, the cellular populations, and the chemistry of the zebrafish and human nervous system are evolutionarily conserved. The nervous system of zebrafish is anatomically divided into the fore-, mid-, and hindbrain, including the diencephalon, telencephalon, cerebellum, and spinal cord (2, 6). The blood–brain barrier (BBB) is structurally and functionally similar to that of higher vertebrates and developed by 3 days post fertilization (dpf) (9, 10).

### Dissection of the Monoaminergic System in Zebrafish

**Figure 4:**
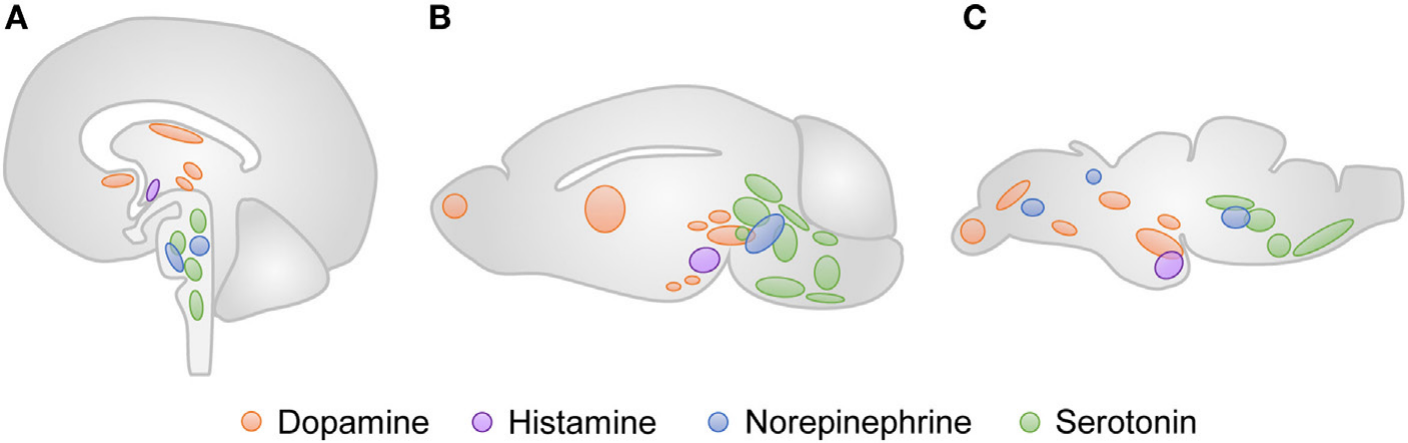
Neuronal clusters that modulate movement in vertebrates. The approximate anatomical location of the dopaminergic (orange), histaminergic (purple), noradrenergic (blue), and serotonergic (green) regions are represented for human **(A)**, rodent **(B)**, and zebrafish **(C)** brains.

Specifically, the monoaminergic system is involved in the adjustment of movement and is predominantly conserved in vertebrates (Figure 4) (11). The tyrosine hydroxylase (TH) is an important marker of catecholaminergic neurons. Two genes, *th1* and *th2*, encode the TH enzyme in zebrafish, and both proteins are highly similar to the mammalian TH (12). The neuronal populations that express TH1 can be found in the olfactory bulb, telencephalon, diencephalon, locus coeruleus, and caudal lobe (13). The neurons that express TH2 are found in the ventral preoptic region, hypothalamus and colocalize with TH1-positive neurons in the diencephalic dopaminergic cluster. The dopamine transporter (DAT) is also detected in this neuronal population (14). The major difference of the zebrafish catecholaminergic system is the absence of dopaminergic neuronal populations in the midbrain. The diencephalic dopaminergic cluster located in the posterior tuberculum of zebrafish has been suggested to be the functional homolog of substantia nigra in mammals (15). Like in mammals, the noradrenergic population is predominantly located in the locus coeruleus of zebrafish. In turn, the catecholamines, dopamine and noradrenaline, are detectable in zebrafish larvae with 5 dpf (16). Zebrafish encode one monoamine oxidase (MAO) with homology to the human MAO-A and MAO-B, and two putative catechol-*o*-methyl transferases (17, 18). The catecholaminergic receptors and transporters are also conserved among vertebrates. Eight subtypes of the dopamine receptor (17), five alpha-2-adrenergic receptors, and one noradrenaline transporter (19) have been identified in zebrafish so far.

The serotonergic and histaminergic systems of zebrafish also present homologies with the corresponding circuits in mammals. The zebrafish serotonergic neuronal groups can be found in the raphe nuclei, pretectum, posterior paraventricular organ, vagal lobe, and reticular formation (20). Particularly, the serotonergic pretectal and paraventricular neuronal populations are not found in higher vertebrates. Three orthologs of the mammalian serotonin receptors have been identified in the genome of zebrafish, along with two genes, slc6a4a and slc6a4b, that encode serotonin transporters (21, 22). The zebrafish histaminergic system consists of a posterior hypothalamic neuronal cluster, from which all histaminergic projections derive (11). Histamine can be detected at 100 hpf (23), and three histamine receptors have been identified in zebrafish (24).

Overall, the expression of monoaminergic proteins and the spatial distribution of monoaminergic neuronal populations are well characterized. Although zebrafish suffered genome duplication, it seems that the distribution of the duplicated proteins is complementary to that observed in mammals. This helps to explain why, despite the consistent differences, the drugs that target transporters, receptors or enzymes involved in the modulation of neurotransmitters have rather conserved effects (25, 26).

## Methods

The aim of this review was to analyze the studies reported to date on the use of zebrafish models for movement disorders, and assess their potential for modeling the human pathologies. To achieve this, we conducted a systematic search of the literature using BiomedCentral, EBSCO host, PubMed/Medline, ScienceDirect, and Web of knowledge, in August and September 2017. The following search strategy was used for each of the five bibliographic databases: Title, abstract, keywords, or topic: (“Parkinson’s disease” OR “Parkinsons disease” OR “parkinsonism”) AND (“zebrafish”); (“progressive supranuclear palsy” OR “supranuclear palsy”) AND (“zebrafish”); (“dystonia” OR “dystonic”) AND (“zebrafish”); (“Tremor” OR “Tremors”) AND (“zebrafish”); (“Tourette’s syndrome” OR “Tourettes syndrome”) AND (“zebrafish”); (“Huntington’s disease” OR “Huntingtons disease”) AND (“zebrafish”); (“Rett syndrome” OR “Rett” OR “RTT”) AND (“zebrafish”). All dates were included in the search criteria. Only published, peer-reviewed studies written in English were considered. The studies with the description of the phenomenology of zebrafish models of movement disorders were included in the review, through scrutiny of the title and abstract of the papers identified during the systematic search. Studies with no description of the pathological mechanisms AND/OR phenotypes of the diseases were excluded from analysis.

For Parkinson’s disease (PD), a total of 39 studies (Figure 2) met the inclusion criteria, from 110 returned by BiomedCentral, 111 by EBSCO host, 221 by PubMed/Medline, 4,015 by ScienceDirect, and 181 by Web of knowledge. For progressive supranuclear palsy (PSP), 1 study fulfilled the inclusion criteria, from 8 returned by BiomedCentral, 2 by EBSCO host, 1 by PubMed/Medline, 207 by ScienceDirect, and 2 by Web of knowledge. For dystonia, 8 studies were in agreement with the inclusion criteria, from 15 returned by BiomedCentral, 7 by EBSCO host, 11 by PubMed/Medline, 363 by ScienceDirect, and 15 by Web of knowledge. For tremor, 1 study met the inclusion criteria, from 23 returned by BiomedCentral, 6 by EBSCO host, 8 by PubMed/Medline, 618 by ScienceDirect, and 9 by Web of knowledge. For Tourette’s syndrome, no study fulfilled the inclusion criteria, from 7 returned by BiomedCentral, 1 by PubMed/Medline, 245 by ScienceDirect, and 2 by Web of knowledge. For Huntington’s disease (HD), 9 studies were in line with the inclusion criteria, from 54 returned by BiomedCentral, 22 by EBSCO host, 31 by PubMed/Medline, 964 by ScienceDirect, and 52 by Web of knowledge. Finally, for Rett syndrome (RTT), 5 studies met the inclusion criteria, from 20 returned by BiomedCentral, 5 by PubMed/Medline, 638 by ScienceDirect, and 13 by Web of knowledge.

## Zebrafish as a Model of Hypokinetic Movement Disorders

Parkinson’s disease and parkinsonism represent the most frequent hypokinetic syndromes. These include akinesia (inability to initiate voluntary movements), bradykinesia (slowness of voluntary movements), gait and balance disturbances (falls), freezing phenomenon (absence or marked reduction of forward stepping during walking), and rigidity (resistance to externally imposed joint movements).

### Parkinson’s Disease

Parkinson’s disease is the most prevalent movement disorder, affecting 100–200 per 100,000 people (27). The etiology of PD is a combination of genetic and environmental factors that, at some point during disease progression, lead to dopaminergic cell loss in the substantia nigra pars compacta and to the accumulation of protein inclusions known as Lewy bodies. These inclusions are primarily composed of the protein α-synuclein (Table 1). Mutations in the alpha-synuclein (*snca*), leucine-rich repeat kinase 2 (*lrrk2*), *vps35*, PTEN induced putative kinase 1 (*pink1*), parkinsonism associated deglycase (*dj-1*), and parkin RBR E3 ubiquitin protein ligase (*parkin*) are associated with familial cases. However, the vast majority of the cases (~90–95%) are affected by sporadic PD. The treatment of motor symptoms aims at replacing dopamine and includes levodopa, among other dopaminergic modulators (Table 1). There are still no disease-modifying agents for PD, but several drugs are under clinical trials.

#### Chemical Zebrafish Models of PD

##### 1-Methyl-4-Phenyl-1,2,3,6-Tetrahydropyridine (MPTP)-Induced Models

Parkinson’s disease is the most studied movement disorder in zebrafish (Table 2). The effects of exposure to MPTP, known to cause loss of dopaminergic neurons and parkinsonism in humans (28), have been studied in zebrafish at all developmental stages (embryonic, larval, and adulthood). MPTP causes specific loss of dopaminergic neurons, a decrease of the dopamine, norepinephrine, and serotonin levels, and motility impairments in zebrafish larvae (29–32). By labeling monoaminergic neurons with GFP, Wen et al. (33) showed that the toxic effects of MPTP are more severe in the dopaminergic neurons located in the posterior tuberculum of the ventral diencephalon, corresponding to the mammalian midbrain dopaminergic neurons (33). Other neuronal clusters, including the serotonergic, also seem to be affected, but in less extent (32). By contrast, MPP+, the metabolite of MPTP, does not affect the serotonergic cluster, indicating a more specific action (32). MPP+ inhibits the multi-subunit enzyme complex I of the mitochondrial electron chain and impairs mitochondrial respiration (34). The subacute exposure to a low dose of MPP+ causes dramatic retrograde mitochondrial transport, before the appearance of neuronal and locomotor changes in zebrafish larvae (35). This suggests that MPP+ damages the mitochondria that are transported back to the cell body for degradation. This mechanism of bioenergetic homeostasis seems to be impaired at higher concentrations of MPP+. The MPTP-induced parkinsonian-like phenotype can be reverted by l-deprenyl (selegiline), an MAO-B inhibitor, and nomifensine, a DAT inhibitor (29, 30, 32). This indicates that the transport of MPTP and its conversion into the active metabolite, MPP+, is mediated by the same mechanisms in zebrafish and in mammals (34). Although selegiline is used as an anti-parkinsonian agent, its restorative effects in MPTP-lesioned zebrafish do not totally mimic the therapeutic effects in PD patients.

**Table 2:** General characteristics of the zebrafish models of hypokinetic movement disorders reviewed.

Disease	Zebrafish model	Pathological hallmarks	Motor phenotype	Responsiveness to pharmacotherapies and disease-modifying therapies	Observations	Reference
Parkinson’s disease	Chemical					
	
	1-Methyl-4-phenyl-1,2,3,6-tetrahydropyridine	Decrease of dopamine levels Loss of dopaminergic neurons	Deficits in evoked swimming response (bradykinesia) Decrease in total distance moved and swimming velocity (bradykinesia) Increase in number and duration of freezing episodes (dyskinesia)	Selegiline rescues neuronal and motor impairments		(29–33, 35–37)
	
	6-Hydroxydopamine	Decrease of dopamine levels Loss of dopaminergic neurons	Decrease in total distance moved and swimming velocity (bradykinesia)	Levodopa + carbidopa rescue motor impairments		(36, 40, 43)
	
	Paraquat	Decrease of dopamine levels	Decrease in total distance moved (bradykinesia)	ND	Only at doses higher than LC_50_ Variable toxic effects	(48, 49, 55, 56)
	
	Rotenone	Decrease of dopamine levels	Decrease in time swimming at high velocity (bradykinesia)	ND		(59)
	
	Cytotoxic metabolite of metronidazole	Decrease of dopamine levels Loss of dopaminergic neurons	Decrease in total distance moved and swimming velocity (bradykinesia) Increase in resting time and decrease in active time	ND	Metabolization driven by nitroreductase, expressed under the control of *dat* promoter	(64)
	
	Titanium dioxide nanoparticles	Loss of dopaminergic neurons	Decrease in total distance moved (bradykinesia)	ND		(65)
	
	Ziram	Loss of dopaminergic neurons	Decrease in total distance moved (bradykinesia)	Apomorphine rescues motor impairments		(74)
	
	Genetic					
	
	β- or γ1-Synucleins knockdown	Decrease of dopamine levels Delayed development of dopaminergic neurons	Decrease in swimming velocity (bradykinesia)	ND		(69)
	
	γ1-Synuclein overexpression	Synuclein aggregates	ND	ND	Developmental defects	(74)
	
	Human α-synuclein overexpression	Synuclein aggregates	ND	ND	100% Lethal at 10 days post fertilization	(75)
	
	Pinkl knockdown	Loss of dopaminergic neurons	Deficits in evoked swimming response (bradykinesia) Decrease in total distance moved (bradykinesia)	ND	Developmental defects	(76, 77, 82)
	
	Parkin knockdown	Loss of dopaminergic neurons	No changes in total distance moved	ND		(87)
	
	DJ-1 knockdown	No loss of dopaminergic neurons	ND	ND		(97)
	
	ΔWD40-LRRK2	Loss of dopaminergic neurons	Decrease in total distance moved (bradykinesia)	Levodopa rescues motor impairments		(101)
	
	LRRK2 knockdown	Synuclein aggregates Loss of dopaminergic neurons	ND	ND	Developmental defects	(102)
	
	FBXO7 knockdown	Loss of dopaminergic neurons	Decrease in swimming velocity (bradykinesia)	Apomorphine rescues motor impairments	Developmental defects	(108)
	
	ATP13A2 knockdown	ND	Decrease in swimming velocity (bradykinesia)	ND	Developmental defects	(109)

Progressive supranuclear palsy (PSP)	A152T-tau overexpression	Increased accumulation of tau and neurofibrillary tangle formation Neuronal death	Deficits in evoked swimming response (bradykinesia)	ND		(115)

*ND, not determined*.

In adult zebrafish, MPTP induces a reduction of the levels of dopamine and norepinephrine, which results in marked decrease of the motor performance (31, 36). MPTP-lesioned adult zebrafish exhibit a significant decrease of the swimming velocity (bradykinesia), an erratic swimming pattern and increase of the freezing episodes (dyskinesia) (37). Strikingly, there is no reduction in the number of dopaminergic neurons, neither the activation of pathways that lead to cell death (31, 36). In mice, the activity of the TH decreases after exposure to MPTP (38). This could in part explain the loss of dopamine and norepinephrine in zebrafish, despite the lack of cellular death. In turn, MPTP could cause a transient loss of function of the dopaminergic neurons, instead of its death. A proteomic analysis in MPTP-lesioned zebrafish revealed altered transcriptional regulation of several genes, including *lrrk2, dj-1, park2*, and *pink1*. In addition, the expression of 73 proteins, some of which associated with neurological pathways, was also changed (37). For instance, the neurofilament light polypeptide-like (NEFL) protein, involved in the glutamatergic and GABAergic signaling in presynaptic nerve terminals, was found downregulated in the brain of MPTP-lesioned zebrafish. As demonstrated in zebrafish larvae, selegiline induces recovery of the PD-like symptoms in MPTP-lesioned adult zebrafish (37). On the other hand, adult zebrafish submitted to an MPP+ injection do not exhibit abnormal phenotype, in contrast to zebrafish larvae (31). MPP+ cannot cross the mammalian BBB (39), suggesting that in adult zebrafish the mature BBB prevents the entry of MMP+ into the central nervous system (CNS). The differences observed in the several developmental stages could result from a lower sensitivity of adult zebrafish to the neurotoxin. The access of MPTP to the brain is very different in each of the developmental stages, which in part could result from the distinct routes of administration adopted. Larvae were exposed to the neurotoxin through the water, because of its extreme permeability, whereas, adults received a single intramuscular or intraperitoneal injection.

##### 6-Hydroxydopamine (6-OHDA)-Induced Models

6-Hydroxydopamine is a hydroxylated compound of dopamine that has been extensively used to induce dopaminergic lesions in rodents (34). Intramuscular injection of 6-OHDA causes a decrease of the dopamine and norepinephrine levels in adult zebrafish (36). Motor impairments are also observed, despite the lack of loss of dopaminergic neurons. By contrast, when administered into the ventral diencephalon of adult zebrafish, 6-OHDA induces significant ablation (>85%) of dopaminergic neurons in the posterior tuberculum and two other dopaminergic clusters, causing bradykinesia (40). The neuronal population in the olfactory bulb is one of the clusters affected. This mimics the phenotype in rats that exhibit olfactory impairments when lesioned with 6-OHDA into the substantia nigra (41). The inability to cross the BBB, explains the different phenotypes observed when 6-OHDA is administered intramuscularly or intracranially in zebrafish. A recovery of the dopaminergic neurons was observed 30 days post-lesion, which can be attributed to the neuro-regenerative capacity of the adult zebrafish brain (42). Therefore, the highly regenerative nature of zebrafish compromises the study of the progressive degenerative process in PD. On the other hand, the intracerebral administration of 6-OHDA revealed to be a laborious and meticulous protocol.

In the zebrafish larval stage, exposure to 6-OHDA in the water induces a decrease in the expression of TH, reduction in the locomotor activity and anxiogenic behavior (43). The locomotor impairments can be rescued by vitamin E, minocycline, and levodopa + carbidopa, the most effective drug used in patients with PD. Vitamin E is also able to normalize the expression of TH, while minocycline attenuates the increase of the expression of TNF-α and *cd11b* mRNA in 6-OHDA-lesioned zebrafish larvae. Vitamin E has antioxidant properties (44) and minocycline has shown anti-neuroinflammatory activity in rodents (45). This suggests that the oxidative stress and inflammatory process induced by 6-OHDA in zebrafish larvae share functional features with mammals.

##### Paraquat-Induced Models

Chronic exposure to pesticides, used in agriculture, has been recognized as a risk factor for development of parkinsonian syndromes. Paraquat induces oxidative stress and cytotoxicity in neurons (46). This herbicide is structurally similar to MPP+ and is associated with increased risk of developing PD (47). In adult zebrafish, the systemic administration of paraquat causes locomotor changes, but no anxiety-like behavior (48). In a different study, anxiolytic and aggressive behavior were observed, but no motor impairments in paraquat-lesioned adult zebrafish (49). While the former mimics the locomotor impairments, the later resembles the anxiogenic behavior in paraquat-lesioned rodents (50–52). The authors suggested that the results may be influenced by the genotype or gender of fish. Adult zebrafish lesioned with paraquat also reveal impairments in spatial memory, a decrease in the ratio of DOPAC/dopamine levels, a decrease in the expression of DAT, lowered mitochondrial viability, and an increase in the expression of antioxidant enzymes (48, 49). TH expression and reactive oxygen species (ROS) levels were unaltered. Despite the lack of cellular hallmarks of PD, the neurobehavioral syndrome in zebrafish is very similar to the one observed in paraquat-lesioned rodents (50, 51, 53). The neurotoxic effects of paraquat in this model are also variable (50, 51, 54).

Alternatively, when added to the water, paraquat seems to induce no parkinsonian-like phenotypes in larvae and adult zebrafish. These exhibit a normal number of dopaminergic neurons, normal behavior, and no developmental defects (31). Instead, when the LC_50_ of paraquat is used, zebrafish larvae exhibit a reduction of the dopamine and serotonin levels, activation of antioxidant and oxidative stress related genes, and distinct macrophage activation and migration (55, 56). In addition, significant increase of apoptotic cells in the head, trunk, and tail, and motor deficits are observed. However, the authors did not rule out general toxicity, leaving doubts about the specificity of the phenotypes observed. In addition, the toxic effects of paraquat seem to have high variability. Zebrafish larvae with 48 hpf exposed to 600 mg/L of paraquat do not show morphological defects, while 0.04 and 100 mg/L of paraquat were determined as LC50 in zebrafish larvae with 18 and 72 hpf, respectively. The successful induction of dopaminergic neuronal death by pesticides in rodents has also been striking. While paraquat is associated with high variability (57), rotenone induces weakly reproducible phenotypes and high mortality rates (58).

##### Rotenone-Induced Models

Exposure to rotenone has been linked to a higher risk of PD (47). In zebrafish, the phenotypes induced by rotenone are incongruent. One study found no cellular or behavioral parkinsonian-like phenotypes in larvae and adult zebrafish exposed to rotenone (31). By contrast, using the same concentration of the pesticide, time of exposure, and route of administration, a different study reported that adult zebrafish have decreased levels of dopamine and TH, deficits in motor function, anxiety and depression-like behavior, and olfactory dysfunction (59). Differences in TH expression and motor performance could be justified by the different protocols used to determine each of the parameters. In one study, the authors determined mRNA expression of TH by *in situ* hybridization and locomotor performance through the mean velocity of swimming. In the other, the authors analyzed TH expression by western blot and motor capacity through monitorization of freezing, swimming at low speed, and swimming at high speed. The difference between rotenone and vehicle treated zebrafish was detectable only at high speed swimming. The phenotypes observed are characteristic of PD patients (60, 61), and some mirror the phenotypes found in rodents (62, 63).

##### Other Neurotoxic Agents

Other less conventional methods have been used to induce dopaminergic neurotoxicity in zebrafish. This is the case of the transgenic line that expresses the reporter cyan fluorescent protein and the nitroreductase enzyme under the control of the *dat* promoter [Tg(dat:CFP-NTR)]. When transgenic larvae are exposed to metronidazole, the nitroreductase metabolizes it into a cytotoxic product that activates the apoptotic pathway and induces dopaminergic cell loss. The process can be monitored in real time, by detection of the reporter protein. Tg(dat:CFP-NTR) zebrafish larvae exposed to metronidazole show a reduction of the number of neurons in several dopaminergic clusters. This coincides with a decrease of dopamine levels and the appearance of locomotor impairments (64). The zebrafish line seems to maintain a persistent decrease of dopaminergic neurons that lasts longer than the other toxin-induced models.

Alternatively, a recent study showed that titanium dioxide nanoparticles (TiO_2_ NPs) cause parkinsonian-like phenotypes in zebrafish larvae (65). Exposure to TiO_2_ NPs induces premature hatching and abnormal development, but no lethality. TiO_2_ NPs accumulate in the brain of zebrafish larvae, resulting in the generation of ROS, loss of dopaminergic neurons, cell death in the hypothalamus and locomotor impairments. An increase in the expression of *pink1, parkin*, and *uchl1* genes was also observed. Surprisingly, the authors also described an increase of the expression of the *α-synuclein* gene. Since zebrafish lack the ortholog of the human α-synuclein, the authors must have wanted to refer to the other two synucleins expressed in zebrafish (66). In rats, TiO_2_ NPs accumulate in the brain, stimulate oxidative stress and inflammatory responses, and cause impairments in the CNS (67). Despite the similarities of the phenotypes observed in zebrafish with the molecular and cellular mechanisms in PD, there is still no association of TiO_2_ NPs with increased risk of PD.

#### Genetic Zebrafish Models of PD

##### Synucleins

Among the several zebrafish genes with homology to human PD genes, an ortholog of the human α-synuclein appears not to be present in the zebrafish genome (68). Instead, zebrafish express three synuclein isoforms, β-, γ1-, and γ2-synucleins, that seem to compensate the absence of α-synuclein. Functionally, the zebrafish γ1-synuclein appears to be the closest to the human α-synuclein. Knockdown of the β- or γ1-synucleins induces motor impairments in zebrafish, which are even more severe when the expression of both synucleins is abrogated (69). Zebrafish lacking both synucleins have an abnormal development of the dopaminergic system, including delayed differentiation of dopaminergic neurons and reduced levels of dopamine. The phenotype can be reverted by the expression of human α-synuclein. Strikingly, the knockdown of the zebrafish β- and γ1-synucleins results in phenotypes that recapitulate the aspects observed in rodents lacking all synucleins (70–73). In zebrafish, overexpression of γ1-synuclein leads to the formation of neuronal aggregates and neurotoxicity, similarly to the human α-synuclein (74). On the other hand, downregulation of γ1-synuclein protects zebrafish from the toxicity of ziram. Exposure to ziram dramatically increases the risk to develop PD. This pesticide causes loss of dopaminergic neurons and impaired swimming behavior in zebrafish (74). Treatment with apomorphin recues the motor impairments. Moreover, CLR01, an inhibitor of amyloidogenic proteins self-assembly, protects zebrafish against ziram-induced neurotoxicity. These data suggest that ziram might induce toxicity on dopaminergic neurons through the formation of γ1-synuclein toxic oligomers. Still, none of the above zebrafish lines exhibit as a severe phenotype as zebrafish overexpressing human α-synuclein. During embryonic development, this line presents neuronal apoptosis, which results in severe deformities and death within 48–72 h (75). The neurotoxic effect of α-synuclein is mediated by the inhibition of the ubiquitin proteasome system and accumulation of α-synuclein. Treatment with CLR01 reduces the aggregation of α-synuclein and neuronal apoptosis, increasing viability. The devastating effects of the overexpression of human α-synuclein may hinder the successful generation of transgenic zebrafish lines. Perhaps, by restricting the expression of the protein to the dopaminergic neurons or to a transient manner could decrease the lethality, while maintaining the pathological mechanisms.

##### PTEN Induced Putative Kinase 1 (*PINK1*)

The gene *PINK1* is implicated in genetic and sporadic cases of PD. Morpholino knockdown of the zebrafish PINK1 ortholog has added evidence to the importance of this gene in the control of oxidative stress and mitochondrial function. Downregulation or total abrogation of the expression of PINK1 results in mitochondrial dysfunction that leads to augmented levels of ROS and activation of the apoptotic signaling pathway in zebrafish (76). The PINK1 null mutant zebrafish line also presents mitochondrial impairments (77). The zebrafish Pink1 influences the expression of other proteins that are critical contributors to the pathogenic process. For instance, the activity of the mitochondrial protein GSK3β is increased and its inhibition, with LiCl and SB216763, partially rescues the phenotypes in PINK1 morphant zebrafish (76). TigarB, the zebrafish ortholog of the human glycolysis and apoptosis regulator Tigar, is also markedly increased in Pink1 null mutants (77). Tigar has been identified as a negative regulator of mitophagy, considered to be crucial in the pathogenesis of early-onset PD (78). The expression of other 177 genes, from the hypoxia-inducible factor (HIF) signaling, TGFβ-signaling, and several key toxicological responses (mitochondrial dysfunction, RAR activation, and biogenesis of mitochondria), is also altered (79). Particularly, the HIF pathway is the most affected pathway in PINK1 knockdown zebrafish. This is known to participate in the regulation of oxidative stress and neuronal differentiation *in vitro* (80, 81).

Whereas, the molecular mechanisms seem to be consistent, the phenotypes induced by the alteration of Pink1 expression in zebrafish vary. PINK1 morphant zebrafish exhibit general developmental delay, severe mispatterning of the axonal scaffold, and moderate decrease of the number of neurons, mainly in the dopaminergic system (76). The phenotype could be rescued by wild-type human *pink1* mRNA. Further supporting these results, PINK1 knockdown zebrafish show changes in neuronal patterning and axonal projections (82). This line also presents mild loss of dopaminergic neurons in the diencephalon, which leads to spontaneous or evoked locomotor impairments. Motor performance could be rescued by the dopamine agonist SFK-38393. Moreover, the expression of exogenous PINK1 rescued all phenotypes. By contrast, a subsequent study reported no morphological or behavioral deficits in PINK1 morphant zebrafish (83). Instead, increased vulnerability to MPTP-induced toxicity was observed. The authors described a reduction in the expression of both, *th1* and *th2* mRNA forms, but normal levels of *dat* mRNA. Although there was a mild decrease in the number of TH-positive neurons in the dopaminergic diencephalic cluster, the normal levels of *dat* suggest that the downregulation of PINK1 may cause a decline in important mRNAs and proteins, instead of neuronal death. Still, the increased susceptibility to MPTP strengthens the importance of PINK1 in oxidative stress. In accordance, the exposure to H_2_O_2_ dramatically increases the expression of *pink1* mRNA in zebrafish, which can be reverted by the antioxidant l-glutathione reduced (84). In *PINK1* mutant zebrafish larvae, the loss of dopaminergic neurons is more evident and accompanied by marked microglial activation (77). In fact, PINK1 deficiency causes different phenotypes in mammals, as well. In humans, mutations *in PINK1* can result in early-onset PD and are associated with mitochondrial dysfunction (85). In turn, *PINK1* knockout mice have a mild phenotype, with no neuronal death, no changes in the levels of striatal dopamine, nor in the number and morphology of mitochondria (86). Interestingly, it seems that PINK1 knockdown zebrafish recapitulate better than mice the human phenotypes.

##### Parkin RBR E3 Ubiquitin Protein Ligase (*Parkin*)

Mutations in PINK1 and Parkin are implicated in mitochondrial dysfunction and seem to share several pathogenic mechanisms in PD. Consistently, Parkin knockdown zebrafish exhibit a phenotype that resembles PINK1 knockdown zebrafish. Abrogation of the expression of Parkin leads to impaired mitochondrial function, specific loss of dopaminergic neurons in the posterior tuberculum, and increased sensitivity to the toxic effects of MPP+ (87). Neither the serotonergic nor the motor neurons are affected, and the extent of dopaminergic loss is not enough to cause behavioral defects. Parkin knockdown zebrafish present two important phenotypes, mitochondrial dysfunction and dopaminergic cells loss, described in PD patients with mutations in *Parkin* (88, 89). Once again, it seems that the zebrafish model mimics better than mice models the pathological mechanisms in humans. *Parkin* knockout mice do not show any robust morphological changes, neither increased susceptibility to MPP+ (90, 91). Notwithstanding, morpholino-mediated knockdown of the zebrafish *Parkin* generates very dissimilar phenotypes. In a different study, no loss of dopaminergic neurons, neither morphological nor behavioral alterations, was observed upon *Parkin* knockdown in zebrafish (92). This result may be the consequence of a partial ablation (around 50%) of Parkin expression. Still, this line maintained the increased vulnerability to stress-induced cell death. Importantly, the authors also described that the overexpression of Parkin in a transgenic zebrafish line protects from proteotoxic stress-induced cell death. Similarly to PINK1, Parkin is suggested to have a protective role in PD (93). In zebrafish, it seems that the presenilin-associated rhomboid-like (PARL) protein is also part of the PINK1 and Parkin pathway. PARL is a component of the mitochondrial membrane involved in mitochondrial morphology and apoptosis. Morpholino knockdown of both zebrafish paralogs, *parla* and *parlb*, results in high mortality, whereas loss of PARLb leads to the mildest phenotype (94). Loss of one of the PARL’s results in mild neurodegeneration and disarranged dopaminergic neurons. Although changes in survival were not reported, generalized cell death was observed. Interestingly, the phenotype can be rescued by human *parl* mRNA and by zebrafish and human *pink1* mRNA. The *PARL* gene has been linked to familial cases of PD. PARL is suggested to be important in the normal trafficking and processing of PINK1 and Parkin in mitochondria (95).

##### *DJ-1* 

Mutations in *DJ-1* are associated with early-onset PD. The inactivation of DJ-1 in zebrafish leads to an increase in the expression of p53 and Bax, but no cellular or morphological changes (96, 97). Moreover, the concomitant knockdown of *DJ-1* and *mdm2*, a negative regulator of p53, results in dopaminergic neuronal death (96). This suggests that p53 may mediate cell loss in the absence of DJ-1. DJ-1 knockdown zebrafish exhibit loss of dopaminergic neurons after exposure to H_2_O_2_ and to the proteasome inhibitor MG132. The phenotype can be prevented with pharmacological inhibition of p53, by pifithrin-alpha (96, 97). This demonstrates that DJ-1 knockdown zebrafish are susceptible to programmed cell death and that DJ-1 may mediate the stress response machinery. In accordance, DJ-1-deficient mice only exhibit dopaminergic cell death after toxin exposure (98). The p53-glycerylaldehyde-3-phosphate dehydrogenase (GAPDH)–Bax pathway has been suggested to be involved in PD (99, 100).

##### Leucine-Rich Repeat Kinase 2 (*LRRK2*)

The phenotypes of LRRK2 morphant zebrafish have been characterized. The first study describing the consequences of the inhibition of the expression of LRRK2, showed embryonic lethality and severe developmental defects, such as brain developmental retardation, in zebrafish (101). A more recent study, described neuronal loss, affecting the dopaminergic system, upregulation of the expression of β*-synuclein, Park13*, and *SOD1*, and β-synuclein aggregation in the CNS (102). The authors also described a wide range of organ abnormalities but did not report such overt toxicity as the former study. On the other hand, the deletion of the WD40 domain of LRRK2 (ΔWD40-LRRK2) causes little impact on embryonic development, in zebrafish (101). Instead, this mutant zebrafish line presents a reduction and disorganization of the axonal tracts, predominantly in the midbrain. In addition, significant loss of the diencephalic dopaminergic neurons and locomotor defects were observed. The phenotype can be rescued by zebrafish and human *lrrk2* mRNA overexpression. The administration of levodopa rescues the motor impairments, but not neurodegeneration, in line with the therapeutic effects in humans. Surprisingly, a subsequent study was not able to replicate the phenotypes described in the ΔWD40-LRRK2 zebrafish line (103). Nevertheless, it has been reported that mutations in the LRRK2-WD40 domain increase neuronal apoptosis under cellular stress (104). In mice, *LRRK2* knockout and G2019S *LRRK2* transgenesis do not induce neuropathological abnormalities, but LRRK2 seems to interfere with normal neurite outgrowth (105, 106).

##### Other PD Genes

Genes implicated in atypical PD, have also been characterized in zebrafish. Park15 is caused by loss of function of the protein encoded by the gene *fbxo7* (107). Morpholino knockdown of the zebrafish Fbxo7 ortholog results in abnormal patterning and loss of dopaminergic neurons, which lead to severe motor impairments (108). Treatment with apomorphine, a dopamine agonist, can revert the locomotor defects. Surprisingly, the human *fbxo7* mRNA failed to rescue the morphological phenotypes. This observation was justified with an atypical timing and localization of expression, as compared with the expression of the Fbxo7 endogenous zebrafish gene. Fbxo7 morphant zebrafish also exhibit developmental defects, as heart deformations, suggesting that Fbxo7 must have an important role in zebrafish development. The zebrafish ortholog of another gene linked to atypical PD, *atp13a2*, has also shown a crucial role during embryonic development. Complete abrogation of the expression of ATP13A2 leads to embryonic lethality, whereas partial knockdown results in abnormal splicing of *atp13a2* mRNA and obvious behavioral impairments (109). The results are in line with experiments in mice, which have also revealed the importance of ATP13A2 during the early stages of embryonic development and neurogenesis (110).

### Effect of Dopaminergic Modulators in Zebrafish

When assessing the validity of an animal model, beyond the pathological hallmarks of the disease, it is important to explore the pharmacologically evoked changes. Studies that report such experiments in zebrafish models of PD are scarce, but there exist some reports exploring the effects of drugs known to modulate movement on healthy zebrafish. For instance, haloperidol and chlorpromazine, two dopamine receptor antagonists, have been tested on zebrafish larvae. The suppression of the dopaminergic signaling by both compounds induces akinetic-like behavior (111). Another study supporting these data showed that the selective dopamine agonists, SFK-38393 and quinpirole, increase motor activity (112). By contrast, the dopamine antagonists, SCH-23390 and haloperidol, decrease motor activity in zebrafish larvae. Interestingly, the non-selective dopamine agonist, apomorphine, and dopamine antagonist, butaclamol, induce biphasic dose-response patterns. This may be attributed to the action of the drugs on multiple dopaminergic receptors. On the other hand, the dopamine antagonists, SCH-23390 and haloperidol, induce different dose-response profiles dependent on the lighting conditions. The authors suggested that the blockade of dopamine receptors in the retinal ganglion cells may have perturbed the adaptation to light/dark conditions. Alternatively, the effects of haloperidol were studied in catalepsy (muscular rigidity). Similarly to rats, it was observed that haloperidol causes the increase of catalepsy in zebrafish (113). This can be reverted by bromocriptine and pramipexole, two dopamine agonists commonly used to improve rigidity. Importantly, this study introduced a new core motor symptom of PD on zebrafish, in alternative to bradykinesia. All the above-mentioned studies demonstrated that the drugs that target the dopaminergic system in mammals elicit similar outcomes in zebrafish, suggesting that the underlying mechanisms that regulate movement are shared by both models. Nevertheless, the demonstration that the phenotypes observed in zebrafish actually result from modulation of the dopaminergic pathway is needed.

Overall, chemical and genetic zebrafish models of PD reproduce several of the biochemical, neurochemical, morphological, and neurobehavioral features of the disease in humans. Importantly, the pharmacological response to drugs used in the clinic is also conserved. The limitations inherent to each model do not seem to surpass the limitations also described in rodents and, in some cases, zebrafish resemble better than rodents, the human features. Finally, the zebrafish genes orthologs to the human genes associated with PD seem to be particularly conserved in terms of sequence and function, as well as, the role of the respective protein in the cellular pathways.

### Other Parkinsonian Syndromes

Progressive supranuclear palsy is a PD-plus syndrome associated with tau neuropathology, which affects about 5–7 per 100,000 people (114). The neuropathological hallmarks include the presence of neurofibrillary tangles (insoluble 4-repeat tau protein) or neuropil threads in the basal ganglia and brainstem. Neuronal loss is diffuse, affecting different neuronal structures (Table 1). Initially, the clinical presentation is heterogeneous but tends to develop to unsteady gait, bradykinesia, unexplained falls, and ocular motor deficits (vertical supranuclear gaze palsy is used to confirm the diagnose). Most of the cases of PSP are sporadic and associated with polymorphisms in the gene that encodes the tau protein, *MAPT*. Mutations in the *MAPT* gene have been identified in several familial cases, as well, but are rarer.

Recently, zebrafish was used to assess the functional and biochemical consequences of a tau variant, p.A152T, identified to increase the risk of PSP in a cohort study (115). The A152T-tau transgenic zebrafish exhibit increased accumulation and phosphorylation of tau, with formation of neurofibrillary tangles (Table 2). The phenotype possibly results from impairments in the proteasome system. The overexpression of A152T-tau also causes neurodegeneration, associated with behavioral deficits in zebrafish. The phenomenology is compatible with the phenotypes observed in A152T transgenic rodent models (116, 117). The use of zebrafish to model PSP is at its beginning and needs further developments. Nevertheless, the data described so far indicate that zebrafish models of PSP may exhibit several neuropathological hallmarks of the disease. Effective pharmacotherapeutic options for PSP are null at the moment, and zebrafish may help to boost the discovery of new drugs.

## Zebrafish as a Model of Hyperkinetic Movement Disorders

Hyperkinetic movement disorders have a more diverse phenomenology and include tremors, dystonia (sustained, repetitive, and patterned muscle contractions), tics (sudden, rapid, repetitive, and non-rhythmic movements), chorea (brief, irregular, abrupt, and non-repetitive movements), and stereotypies (repetitive or ritualistic movements), among others.

### Dystonia

Dystonia is a common and clinically heterogeneous disorder, which can be manifested as an isolated clinical condition (primary dystonias) or associated with other neurological disorders (secondary dystonias) (118). The cause is diverse and includes genetic and environmental factors (Table 1). This phenomenon is believed to result from malfunction of the basal ganglia and, consequently, abnormal plasticity of the sensorimotor cortex (119). The pharmacotherapy for dystonia is solely symptomatic, mostly empirical, and adapted to each case (Table 1) (120). In the last 5 years, zebrafish have been used to understand the mechanisms of dystonia.

#### Genetic Zebrafish Models of Dystonia

The most common cause of early-onset primary dystonia is a mutation in the *TOR1A* gene (121). In zebrafish, the ortholog gene, *tor1*, is not essential for early development of the motor system. The morpholino-mediated knockdown zebrafish line presents normal viability, morphology, development, and behavior (Table 3) (122). Zebrafish TOR1 may have an important role in later events, but the transient effect of morpholino-mediated knockdown did not allow to confirm this fact. Accordingly, in *TOR1A* knockout mice, the first phenotypic abnormalities are only observed in a later developmental stage (123). Other genes implicated in dystonia are involved in neuronal development and brain maturation in zebrafish. For instance, dystonia in early childhood can be caused by an autosomal recessive mutation in the pantothenate kinase 2 (*PANK2*) gene (124). Morpholino knockdown of the zebrafish ortholog perturbs the neuronal development and brain morphology and induces hydrocephalus (125). The phenotype can be rescued by pantethine and coenzyme A. To further test the implication of mutations identified in the *col6a3* gene of subjects with primary dystonia, a study found that morpholino knockdown of the zebrafish ortholog gene causes deficits in axonal outgrowth (126). The authors suggested that Col6a3 may participate in the structural organization of neurons. Therefore, its disruption can hamper the establishment of correct neuronal circuitries and synaptic remodeling processes, during brain development and maturation. Finally, knockdown of the ortholog of the human *Atp1a3* leads to brain ventricle dilation and depolarization of Rohon–Beard neurons in zebrafish (127). Although response to tactile stimuli and motility are altered, the dopaminergic neurons seemed to be unaffected. Mutations in the *Atp1a3* are implicated in rapid-onset dystonia parkinsonism (RDP) (128). Ventricle dilation is not observed in patients with RDP, but numerous symptoms reported in dystonic patients suggest the involvement of the somatosensory system. In turn, the depolarization of Rohon–Beard neurons in zebrafish is indicative of altered neuronal excitability, also described in rats (129).

**Table 3:** General characteristics of the zebrafish models of hyperkinetic movement disorders reviewed.

Disease	Zebrafish model	Pathological hallmarks	Motor phenotype	Responsiveness to pharmacotherapies and disease-modifying therapies	Observations	Reference
Dystonia	TOR1A knockdown	No loss of dopaminergic neurons	No changes in swimming velocity or active time	ND		(122)
	
	PANK2 knockdown	ND	ND	ND	Developmental defects	(125)
	
	COL6A3 knockdown	ND	ND	ND	Deficits in axonal outgrowth	(126)
	
	ATP1A3 knockdown	No loss of dopaminergic neurons Altered neuronal excitability	Deficits in evoked swimming response	ND		(127)
	
	SLC30A10 and SLC39A14 mutation	Impaired dopaminergic and GABAergic signaling	Decrease in total distance moved and locomotor activity	Chelation therapy and iron supplementation reverses manganese accumulation and motor impairments		(130, 131)
	
	DBT mutation	Dysregulation of glutamate signalling	Deficits in evoked swimming response Absence of C-bending evoked swimming response (severe dystonia)	ND		(132)
	
	Matrine and sophocarpine	ND	Decrease in total distance moved and swimming velocity Decrease in locomotor activity	ND	Developmental defects	(133)

Chorea in Huntington’s disease	HTT knockdown	Neuronal apoptosis Decreased brain-derived neurotrophic factor expression	ND	ND	Neurodevelopmental abnormalities	(138–140)
	
	HTT polyQ expanded fragments overexpression	Insoluble protein inclusions	ND	ND	Developmental defects Increased apoptosis	(147, 148)
	
	mHTT-ΔN17-97Q overexpression	Neuronal death Intranuclear protein aggregates	Abnormal swimming	ND		(149)

Stereotypies in Rett syndrome	MeCP2 mutation	ND	Increased duration and number of contractions in evoked swimming response Decrease in locomotor activity and swimming velocity	ND		(157)
	
	MeCP2 knockdown	Abnormal axonal branching and outgrowth	Decrease in locomotor activity Deficits in evoked swimming response	ND		(166, 167)

Essential tremor	TENM4 knockdown	Abnormal axonal branching and outgrowth	ND	ND		(171)
	
	Human mutated TENM4 overexpression	Abnormal axonal branching and outgrowth	ND	ND		(171)

*ND, not determined*.

In metabolic disorders, the risk to develop dystonia increases when the manganese homeostasis is compromised. In zebrafish, the mutation of the orthologs of the human manganese transporters, *slc30a10* and *slc39a14*, results in manganese accumulation in the brain (130, 131). This leads to impaired dopaminergic and GABAergic signaling. Changes in the swimming pattern are also visible upon exposure to manganese. The phenotype can be reverted by chelation therapy and iron supplementation, currently used in the clinical practice. Maple syrup urine disease is another metabolic disorder, caused by mutations in the dihydrolipoamide branched-chain transacylase E2 (*DBT)* gene, which can result in severe dystonia. In zebrafish, disruption of the ortholog gene results in elevated levels of branched-chain amino acids (BCAA) (132). This phenomenon is also evidenced in mammalian models and patients. The increase of BCAA leads to the dysregulation of the neurotransmitter glutamate in the brain and spinal cord of zebrafish, which probably contributes to the progressive aberrant motility behavior evidenced. This phenotype was suggested to represent severe dystonia in zebrafish larvae.

#### Chemical Zebrafish Models of Dystonia

Zebrafish are sensitive to neurotoxic drugs that may cause dystonia. Matrine and sophocarpine, two drugs responsible for poisoning juvenile and infant patients, induce growth retardation in zebrafish (133). Exposure to these drugs also led to changes in spontaneous movements and locomotor performance. The authors suggested that this phenotype results from the neurotoxic effects of the drugs but did not show evidence of neurotoxicity.

In summary, it appears that the heterogeneous nature of dystonia can be reproduced in zebrafish. Zebrafish offers an excellent opportunity to understand the pathogenic mechanisms behind the vast number of genetic and environmental factors linked to dystonia. Nevertheless, this heterogeneity seems to result in a large number of studies, with minor characterization and no consolidation of the observed phenotypes. This may undermine a correct judgment about the validity of zebrafish as a vertebrate model of dystonia. Most certainly, further studies are essential.

### Chorea in Huntington’s Disease

Chorea is the most common symptom of HD, which affects around 4–10 per 100,000 people in the western world (134). HD is an autosomal-dominant neurodegenerative disorder, which results from abnormal expansion of the CAG repeat in the huntingtin (*HTT*) gene (polyglutamine disease) (Table 1). The pathogenesis of HD results from the toxic effects of the mutant HTT RNA and protein, HTT aggregation (intranuclear inclusions of abnormal HTT are pathological hallmark) and impairments in protein homeostasis and clearance. These events lead to the death of GABAergic medium spiny neurons in the striatum. Chorea can be improved by tetrabenazine, and the therapeutic effects of other drugs are only empirical (120). There is currently no disease-modifying treatment for HD.

#### Genetic Zebrafish Models of HD

The zebrafish ortholog of the human HTT only encodes four glutamines, compared with up to 35 in humans (135). The zebrafish HTT protein is essential for iron, lipid, and cholesterol homeostasis (136, 137), energy metabolism (136), and brain development (138–140). Its knockdown leads to diverse phenotypes, including hemoglobulin deficiency (136), neuronal apoptosis in the midbrain and hindbrain (138, 139), neurophysiologic abnormalities (138–140), decreased expression of brain-derived neurotrophic factor (BDNF) (138), deficient formation of neural tubes and cell adhesion (140), increased activity of metalloproteinases (ADAM10 and Ncadherin) (140), and severe reduction in cartilage biogenesis (Table 3) (137). Consistently, patients with HD exhibit deficits in iron homeostasis (141, 142), energy metabolism (143), BDNF expression (144), and metalloproteinases activity (145). As observed in *HTT* knockout mice, the complete abrogation of the expression of zebrafish HTT results in embryonic lethality (138). In fact, the mouse models of HD either lack overt phenotypes or exhibit premature death (146). This makes the HTT morphant zebrafish a valuable alternative model to study the cellular function of HTT and its role in the pathological mechanisms of HD. Nevertheless, morpholino-mediated knockdown may result in variable phenotypes and must be considered cautiously.

Zebrafish lines expressing normal and expanded polyglutamine (polyQ) fragments of HTT have been reported (147, 148). In these lines, the misfolding, oligomerization, aggregation, and toxicity of the polyQ fragments are length dependent, in a manner similar to that observed in other animal models and in patients. Zebrafish embryos overexpressing fragments with more than 35Q repeats (mutant form) display insoluble protein inclusions and increased apoptosis (148). This leads to abnormal morphology and development. Strikingly, apoptosis can be detected in cells with no visible inclusions, suggesting that the oligomeric forms of the HTT may be the toxic components. The ubiquitous expression of the polyQ proteins may contribute to the severe phenotype observed. In alternative, it would be interesting to observe the effects of polyQ expression restricted to the CNS. The toxic effects and aggregation of the mutant fragments can be suppressed either by the chaperones Hsp40 and Hsp70 or by the ubiquitin ligase C-terminal Hsp70-interacting protein, resembling other HD models.

Remarkably, the expression of a mutated polyQ fragment lacking the 17 amino acids of the HTT N-terminal tail (mHTT-ΔN17-97Q) elicits toxicity only in neuronal cells of a transgenic zebrafish line (149). Particularly in neurons, mHTT-ΔN17-97Q fragments rapidly form massive intranuclear aggregates. This demonstrates that the neuronal cells have lower capacity to maintain the proteostasis of the expanded polyQ fragments. Moreover, the mHTT-ΔN17-97Q fragments tend to aggregate more and induce a more severe phenotype than the polyQ fragments with an intact N17 terminal. In mice, the expression of the *htt-97Q* gene lacking the N17 causes dramatic accumulation of nuclear mutant HTT aggregates and a robust striatal neurodegeneration that leads to adult-onset movement disorder (150). These results suggest that the N17 portion of the HTT protein substantially prevents the translocation of mutant HTT into the nucleus and plays an important role in the molecular mechanisms of the pathogenesis of HD. mHTT-ΔN17-97Q transgenic zebrafish are the first to recapitulate one of the pathological hallmarks of HD.

#### Chemical Zebrafish Models of HD

The administration of quinolinic acid (QA) into the striatum of adult rodents has been used to induce brain injury that replicates HD (151). Interestingly, while inducing injury, this excitotoxin also stimulates the subventricular neurogenesis zone and neuroblast migration (152, 153). This observation encouraged Skaggs and colleagues (154) to lesion the telencephalon of adult zebrafish with QA and study its neuronal effects. The QA induces cell death and microglial infiltration in the zebrafish CNS (154). However, it also stimulates cell proliferation and neurogenesis that results in total repair of the damage. The authors suggested that this zebrafish model is a powerful tool to study neuronal regeneration in an adult vertebrate and to test potential disease-modifying therapies. Still, the neurogenesis process in the CNS of adult zebrafish is very different from mammals, which might render the translation of the observations puzzling.

In general, the zebrafish HTT shares several important functions with the mammalian ortholog. Zebrafish lines that overexpress mutant HTT are proving to be useful to model HD. Nevertheless, there is only a shallow description of the motor phenotypes, and the responsiveness to pharmacotherapies still needs to be tested on these models.

### Stereotypies in Rett Syndrome

Rett syndrome is a non-neurodegenerative disorder, which affects 1 in 10,000 females by the age of 12 (155). Caused by either nonsense or missense mutations in the methyl-CpG-binding protein 2 (*MECP2*) gene, RTT is characterized by hand stereotypies (Table 1). MeCP2 is a nuclear protein that recognizes DNA methylation to, presumably, regulate gene expression and activation. The patients with RTT, exhibit abnormally small and densely packed neurons, with reduced dendritic complexity and synaptic density. At the cellular level, alterations in different signaling and homeostatic pathways are reported, along with mitochondrial dysfunction and oxidative stress. The options available for the treatment of RTT are currently limited, but several compounds are under clinical trials. These include modulators of neurotransmitters or regulators of cellular metabolism and homeostasis (Table 1).

#### Genetic Zebrafish Models of RTT

In mice, null mutations in MeCP2 drastically reduce lifespan (156). By contrast, zebrafish carrying a null mutation in MeCP2 show normal viability and fertility (157). Instead, MeCP2-null zebrafish exhibit clear motor impairments at early developmental stage (Table 3). These include spontaneous and sensory-evoked motor anomalies, and defective anxiety-like behavior. This zebrafish line has a nonsense mutation in the methyl-CpG binding domain (mecp2^Q63X^), crucial for protein function. The authors suggested that the modest phenotype observed may result from a compensatory mechanism triggered by other proteins belonging to the MeCP2 family or from gene duplication in zebrafish. On the other hand, the studies exploring thigmotaxis in mouse models of RTT have yielded confounding results, some of them contradictory to the ones observed in zebrafish. The authors advocated that the complexity of the neuronal circuitry in mice may have hampered the interpretation of the results in behavioral tests. Later proteomic analysis in the MeCP2-null zebrafish line revealed changes in the expression of proteins critical for energy metabolism, balance of redox status and muscle function (158). This is in line with the reported in RTT patients and experimental mouse models (159–164).

Additional studies in zebrafish confirmed the essential role of MECP2 in neuronal differentiation (165), axonal branching of primary motor neurons (166), and peripheral innervation of sensory neurons (167). The studies further proved that MeCP2 regulates the expression of several cell differentiating factors (Id1–Her2 axis), BDNF and axonal guidance cues (such as Sema5b and Robo2). The indirect disruption of the expression of these genes is involved in RTT-like phenotypes. Accordingly, downregulation of MeCP2 induces a decrease in motor activity and impairments in the sensory function of zebrafish, as observed in mice with partial loss of MeCP2 (168).

All these studies strengthened the notion that the zebrafish MeCP2 is crucial for the regulation of gene expression and activation. Therefore, Mecp2-deficient zebrafish have several phenotypes, reminiscent of the phenomenology observed in mouse models of RTT and in patients with RTT. The normal lifespan in MeCP2-null zebrafish may enable a more profound characterization of the pathophysiological dynamics of RTT and the screening of new drugs.

### Other Hyperkinetic Syndromes

Tremor is a rhythmic oscillation of a body part. Besides resting tremor in PD, this phenomenology is mostly common in the neurologic disorder essential tremor (ET) (120). Progressive action tremor is the classic feature of ET. The most effective drugs for the treatment of ET are propranolol and primidone (Table 1) (169). However, these drugs induce highly variable therapeutic effects and are associated with several adverse effects. The anticonvulsant, topimarate, the GABA agonist, gabapentine, and several benzodiazepines have also shown to improve tremor. The pathological mechanisms and etiology of ET are highly heterogeneous (170). In addition, these are difficult to identify, because ET is commonly a comorbidity. Several genetic and environmental risk factors have been suggested, but none was consistently confirmed in larger cohort studies. This renders the discovery of effective pharmacologic treatments particularly difficult.

Zebrafish may be a practical choice to unravel some of the pathological mechanisms and risk factors implicated in ET. This model has been used to explore the physiological role of *TENM4* and the pathological effects of mutations identified in the *TENM4* of families with ET. Morpholino knockdown of the *TENM4* zebrafish ortholog results in a modest reduction of myelination and aberrant extension, branching and architecture of small axons in the CNS of zebrafish (Table 3) (171). Zebrafish expressing mutated human *TENM4* mRNA show a similar phenotype. These observations are concordant with studies in other animal models. Since 2015, no other study reported the generation of a zebrafish model of tremor, possibly because of the limited knowledge about the nature of ET.

Perhaps for the same reason, at the time of this review, no zebrafish model of Tourette’s syndrome has been reported. There is limited understanding of the etiology of this multifactorial syndrome (Table 1), because several genetic variants and mutations, and non-genetic determinants are implicated. Moreover, these are not exclusive of the disease. This heterogeneity results in a complex and variable neurological and clinical phenomenology. Tourette’s syndrome is typically manifested by various motor and phonic chronic tics (172). The cortico–striato–thalamocortical circuitry is potentially impaired, but the specific neuronal pathway(s) involved remain unknown. Neuroleptic drugs and the monoamine depleting drug, tetrabenazine, are the most effective for tic suppression (Table 1). There exist different rodent models of tics, but their validity is debatable. The inability to assess several human symptomatic features, such as premonitory sensation, is an important limitation of these animals. While the same skepticism can apply to zebrafish, zebrafish models of Tourette’s syndrome may help to elucidate the interplay between genetic and non-genetic risk factors. Ultimately, this may provide valuable clues about the neuropathology of this syndrome.

## Discussion

Zebrafish have been extensively used in the study of the CNS. More recently, the use of zebrafish as a model of human brain diseases and for drug discovery has increased (2, 173). Here, we review several zebrafish models of movement disorders and discuss their translational value. Overall, these models exhibit conserved biochemical and neurobehavioral features. In retrospect, many advantages can be named, but pitfalls must also be highlighted.

First, all studies used zebrafish during embryonic, larval or young adult stage. Practical reasons can justify the use of zebrafish at these developmental stages. For instance, at embryonic and larval stage zebrafish are more permeable, enabling the delivery of drugs through the water. In higher vertebrates, some of the neurotoxic agents used to model movement disorders have to be delivered directly in the brain (174). This approach increases variability between animals and is much more invasive, resulting in the death of some animals. Furthermore, the time lapse between drug administration and appearance of the first phenotypes is much longer in rodents than in zebrafish. Nevertheless, the use of zebrafish larvae is not as accurate as the use of adult zebrafish, where the BBB is fully functional and better mimics the mammalian physiology. The use of zebrafish at early developmental stages also allows to explore the function of specific genes during system development and maturation. However, it must be considered carefully when extrapolating the phenotypes observed to chronic and late-onset disorders. Moreover, the mutant and transgenic lines described here were characterized very early during development, and whether these lines display any pathology in adulthood was not reported. Inevitably, the brain of zebrafish is more complex at adulthood and may mimic more accurately the physiologic features of the mammalian brain.

In turn, morpholino-mediated knockdown is extensively used, because it is a practical tool to reveal the phenotypes induced by downregulation of a gene in zebrafish, but it also has many pitfalls. The extension of abrogation of the expression of the gene can vary drastically, depending on the knockdown strategy and efficiency. Zebrafish possess duplicates of several genes, which can result in a differentiated regulation of gene expression and different phenotypes. In addition, morpholino-mediated knockdown has an acute and transient effect, which does not mimic the chronic effects of downregulation of a gene. On the other hand, non-specific and off-target effects are common, most of the studies lacked the right controls to rule out these effects and, therefore, could not exclude that other systems could be affected. Finally, the knockdown of a gene may not be suited to model some diseases. This is the case of HD, as loss of function of the HTT protein alone does not seem to lead to the disorder (175). The genetic manipulation of zebrafish is particularly easy and should be more exhaustively explored. The mutated or transgenic zebrafish lines develop rapidly, which is an advantage when compared with the time-consuming development of rodent models. When developing transgenic lines, neuronal promoters should be used, to prevent overt toxicity of the transgene. This has been observed for zebrafish lines that overexpress human α-synuclein or HTT polyQ fragments with non-specific promoters (75, 148). In rodents, neuronal promoters are commonly used to restrict the expression of the proteins to the CNS (176).

Another limitation of most of the studies is the restriction of the characterization of the zebrafish model to a single cellular biomarker or behavioral parameter. For instance, most of the studies in zebrafish models of PD determined the expression of TH to assess the integrity of the dopaminergic system, but DAT would be a more specific biomarker for this neuronal population. The zebrafish proteomics is conserved, which allows the use of commercially available biomarkers of other species. Several of these markers have already been tested on zebrafish targets, and many present similar reactivity. In turn, almost all studies limited the evaluation of behavioral changes to total locomotor activity and/or speed, which does not represent the multi symptomatic nature of movement disorders. The assessment of these parameters in zebrafish is a sound strategy, because they are related to the parameters used in rodents to depict bradykinesia. Nevertheless, zebrafish possess a diverse repertoire of behaviors with homology to humans, which have been cataloged and can be easily explored by experimenter-independent behavioral tracking systems (177). Several behavioral tests have been optimized to evaluate motor performance, motor coordination, balance, escape responses, exploratory behavior, reward/punishment-related behavior, learning, memory, social interaction, and aggressive or anxious behaviors (178–183). It is now crucial to overview the array of behavioral tests available for zebrafish, as it has been systematically done for rodents (184, 185).

Furthermore, similar to other models, a deeper characterization of zebrafish will certainly improve the validity of this model system. Regardless the differences between the zebrafish brain and mammalian brain, homologous functions have been attributed to different neuronal regions of each vertebrate. This is the case of the zebrafish diencephalic dopaminergic region (15). However, other neuronal components and physiologic events that modulate movement are still highly unknown. It would be relevant to understand the cerebral components of the zebrafish brain that correspond to the constituents of the mammalian basal ganglia–thalamocortical circuits and to investigate how they are interconnect. Is there a direct and indirect pathway-like system in zebrafish? How do zebrafish control movement features like velocity or direction? Perhaps, to dissect these processes in simpler brain circuitries, as the zebrafish ones, will help to understand more complex mechanisms in mammals. For instance, the small size of the zebrafish brain is useful for three-dimensional mapping of brain structures. With the up-to-date microscopic techniques, whole-brain neuronal connectivity can be easily performed in zebrafish and reveal anatomical relationships, that in larger brains may not be as facilitated. Another particularity that should be further explored is the regenerative capacity of the zebrafish CNS. Several neuronal proliferating sites were identified in the zebrafish brain as compared with two found in the mammalian brain (186). Notwithstanding, many of the molecular and cellular factors that drive regeneration in the brain of adult zebrafish are poorly understood and yet unknown. This may difficult the translation of disease-modifying drugs identified using zebrafish.

Finally, the distinctive and reproducible behaviors of zebrafish exposed to certain neuroactive drugs are a powerful evidence of the conserved functional properties of the neuronal circuitries in vertebrates (187–189). In addition, it highlights the translational value of zebrafish. To improve the validity of this model, it is now important to explore the mechanisms triggered by these drugs on the zebrafish targets. This will increase our understanding of the zebrafish neuronal modulation, and most importantly, enlighten the pharmacodynamic properties of the compounds in zebrafish. In fact, the factors that influence the pharmacodynamic and pharmacokinetic properties of drugs in zebrafish are poorly understood. For instance, previously, it has been assumed that zebrafish totally absorb and distribute through the system the small molecules present in the water (190). However, a recent review has suggested that the absorption of chemicals, as well as distribution through the BBB in zebrafish is comparable to mammals (191). Therefore, the chemical properties of the compounds should be considered when extrapolating concentrations between these two models. The metabolism and excretion of drugs in zebrafish are also difficult to predict. Despite zebrafish have important metabolic enzymes also found in mammals (192), these are not fully characterized. In addition, several differences in the metabolism of chemicals have been reported (193). The compounds used in the clinic are extensively characterized and are particularly adequate to investigate the differences of these compounds on fish and mammals. This would also create a basis to more precisely extrapolate doses between both models.

Probably, a key pitfall in the discovery of new drugs in zebrafish is the absence of general guidelines to calculate mammalian equivalent doses from zebrafish doses. While between mammalian models there exist established formulas (194, 195), the dose extrapolation from zebrafish to mammals is still empirical nowadays. Moreover, much of the existing literature has omitted this rationale (196–199). The only way to understand this rational is by increasing the number of studies where the properties of drugs in zebrafish are translated to mammals. Until now, it seems that only a minority has reached this far. It is not clear dough, whether there is little interest on these drugs, or they actually did not show the same properties in rodents and were not reported. The translation of the discoveries will allow the elaboration of a meta-analysis where the effective doses in zebrafish and mammals can be compared. Ultimately, it would boost the generation of formulas that rule dose extrapolation from zebrafish to different mammalian models and increase the validity of zebrafish models.

## Conclusion

This review underscores the strengths and limitations of the zebrafish models of movement disorders developed to date. Importantly, it raises awareness that zebrafish can mimic the phenomenology of different movement disorders but needs further characterization. To date, there are a substantial number of studies reporting the use of zebrafish as a model for PD. However, for other movement disorders, this number is still limited. Considering the pathological hallmarks, motor phenotypes, and responsiveness to pharmacotherapies, from the seven movement disorders reviewed here, zebrafish models have only been fully characterized in the context of PD. Notwithstanding, the use of zebrafish to model human disorders dates back to the beginning of the twenty-first century, which compared with the 500 years of use of rodent models, is at embryonic stage. This difference of half a century may explain the skepticism that still exists about the use of zebrafish as an animal model of human diseases. The number of studies reporting the use of zebrafish as an animal model is growing and, therefore, the analysis of the pros and cons of the use of this vertebrate model for drug discovery is important.

This study also provides a comprehensive assessment of the methodologies adopted and emphasizes that most of the limitations are inherent to it. Many techniques are available to surpass these limitations and generate consistent and well-characterized models of movement disorders. With the next-generation sequencing, to couple genomic approaches with *in vivo* studies will not only improve our ability to understand the pathogenic mechanisms of complex diseases, as movement disorders but also precipitate the discovery of novel drugs for these disorders. Zebrafish should be considered a practical and inexpensive tool for this approach, provided that, as with any other non-mammalian model, the potential molecules selected in it are further validated by studies in mammals. It is not expected of zebrafish models of movement disorders to fully recapitulate such complex human phenomenology. Even mammalian models have their flaws and do not precisely mimic the symptomatology evidenced in patients. Furthermore, several compounds selected using the traditional models have also failed to demonstrate therapeutic effects in humans. Finally, it is vital to create a comprehensive correlation between zebrafish and mammalian models and, ultimately, be able to translate the findings to humans.

Overall, while it is already evident that zebrafish models of movement disorders share many cellular and physiologic mechanisms with mammalian models and patients, this model is still showing its usefulness for drug discovery. It was not until 2011 that a positive hit from a zebrafish-based drug screening entered phase I clinical trials (200). The usefulness of zebrafish to model human diseases will only be unquestionable when a drug selected in this model proves efficacy in human patients. Meanwhile, the use of zebrafish to study movement disorders will certainly result in a better understanding of their mechanisms and, hopefully, in the discovery of better therapies.

## Author Contributions

RV and JF conceived and outlined the study. RV wrote the first draft. TO and JF did critical revision.

## Conflict of Interest Statement

RV was affiliated with TechnoPhage SA, under the scope of a PhD student scholarship financed by FCT. All other authors declare no conflicts of interest.
